# Validation of Wearable Sensors during Team Sport-Specific Movements in Indoor Environments

**DOI:** 10.3390/s19163458

**Published:** 2019-08-07

**Authors:** Mareike Roell, Hubert Mahler, Johannes Lienhard, Dominic Gehring, Albert Gollhofer, Kai Roecker

**Affiliations:** 1 Institute of Sports and Sports Science (IfSS), Albert-Ludwigs-University Freiburg, Schwarzwaldstraße 175, 79117 Freiburg im Breisgau, Germany; 2 Sport-Club Freiburg e.V., Schwarzwaldstraße 193, 79117 Freiburg, Germany; 3 Institute of Applied Health Promotion and Exercise Medicine (IfAG), Furtwangen University, Robert-Gerwig-Platz 1, 78120 Furtwangen, Germany

**Keywords:** player monitoring, inertial measurement unit, wearables, sensor fusion, indoor team sports

## Abstract

The aim of this study was to determine possible influences, including data processing and sport-specific demands, on the validity of acceleration measures by an inertial measurement unit (IMU) in indoor environments. IMU outputs were compared to a three-dimensional (3D) motion analysis (MA) system and processed with two sensor fusion algorithms (Kalman filter, KF; Complementary filter, CF) at temporal resolutions of 100, 10, and 5 Hz. Athletes performed six team sport-specific movements whilst wearing a single IMU. Mean and peak acceleration magnitudes were analyzed. Over all trials (*n* = 1093), KF data overestimated MA resultant acceleration by 0.42 ± 0.31 m∙s^−2^ for mean and 4.18 ± 3.68 m∙s^−2^ for peak values, while CF processing showed errors of up to 0.57 ± 0.41 m∙s^−2^ and −2.31 ± 2.25 m∙s^−2^, respectively. Resampling to 5 Hz decreased the absolute error by about 14% for mean and 56% for peak values. Still, higher acceleration magnitudes led to a large increase in error. These results indicate that IMUs can be used for assessing accelerations in indoor team sports with acceptable means. Application of a CF and resampling to 5 Hz is recommended. High-acceleration magnitudes impair validity to a large degree and should be interpreted with caution.

## 1. Introduction

Monitoring performance and workloads in team sports is vital when developing and evaluating training programs adapted to the intermittent nature of these sports [1]. The gold standard techniques to capture human movement include three-dimensional (3D) motion analysis systems and force platforms. Due to complex operational demands and their fixed installation in a laboratory, such systems cannot be used to directly measure individual performance in the field. Instead, sport practitioners need more feasible methods that can be applied during training sessions or competitions to quantify overall locomotion of multiple players at the same time. GPS-based technology is, for instance, widely used in outdoor sports but cannot be used in indoor environments due to satellite signal interferences. Therefore, Inertial Measurement Units (IMUs) containing an accelerometer, gyroscope, and occasionally magnetometer have been proposed as alternatives for performance evaluations in indoor sports [2,3,4]. Sufficient concurrent validity of these measurement systems is essential to yield sensitive indicators of an athlete’s power output. Potential sources of error can arise from either internal factors related to IMU specificities or external factors that are directly associated with the execution of these sports [5,6,7].

Internal factors affecting an IMU’s accuracy include the complex process of sensor fusion that is required to determine the device’s orientation with respect to global space [8]. Team sports are intuitively observed and described in an earth-related coordinate system, which can be defined by the z-axis running parallel to earth’s gravitational acceleration and the horizontal axes being in accordance with the field’s dimensions. In contrast, IMUs typically record data within a local coordinate system whose axes are defined by placement within the tracking device. Before interpreting IMU data, an alignment between both coordinate systems is required to eliminate gravity from the sensor’s readings.

Combining single inertial sensors by sensor fusion allows the global orientation to be calculated between both coordinate systems. Fusion can be performed by gyroscope integration, vector observation, Kalman filtering (KF), or Complementary filtering (CF) [9]. The first two algorithms are unsuitable for sport-specific settings since gyroscope integration suffers from integration-induced drift, and the accuracy of the vector observation is restricted to slow movement velocities [9,10]. In general, either KF or CF techniques are applied for orientation estimation during human motion [11,12]. While KF gives a probabilistic calculation of state estimations, CF relies on analysis in the frequency domain of the respective sensor readings. Direct comparisons of both filters has found a better accuracy of CF under simulated orientations. This is probably because CF has a faster rate of convergence, which allows it to follow the true signal accurately, even during momentary pauses in motion [6,13]. However, pre-processed parameters provided within the proprietary software of tracking devices typically rely on KF-based algorithms. Even though the validity of these parameters has previously been examined, current research is mostly restricted to evaluating the resultant acceleration magnitude [14,15]. More detailed analysis in the horizontal and vertical movement planes is not typically provided by manufacturers, even though this is crucial for comprehensive and meaningful analysis of athletes’ locomotor demands during sports. Relying on manufacturer-independent algorithms for raw data processing could improve the analysis of locomotor demands and increase the transparency of data treatment for other investigators.

Another internal factor influencing the accuracy of wearable tracking devices is the sampling frequency. Commercially-available IMUs typically operate at a sampling frequency of 100 Hz in indoor environments, while ranges of 5–15 Hz are standard for GPS devices. The validity of GPS signals has been shown to depend also on the sampling rate itself, whereas no further increase in accuracy was observed for frequencies >10 Hz [16,17,18]. To our knowledge, the influence of temporal resolution on IMU measurement error has not yet been investigated, even though findings from GPS research suggest a sufficient accuracy at resolutions of 5–10 Hz.

Previous studies investigating the concurrent validity of IMUs primarily identified external rather than internal factors affecting validity, with respect to manufacturer-provided parameters [14,15,19]. Findings clearly point towards the intensity of motion as well as the performed movement task itself affecting the IMU’s concurrent validity [14,19], with higher errors being found for high-intensity movements such as sprinting, jumping, and tackling. However, no study has yet investigated whether additional upper body motion evoked by ball handling might influence IMU accuracy, even though ball possession is a crucial part of team sports. Therefore, this study aims to evaluate the concurrent validity of CF- and KF-derived acceleration values measured by a commercially-available IMU during team sport-specific movements. A second goal of this study is to determine IMU-specific internal factors, such as a sensor fusion algorithm and temporal resolution, as well as sports-related external factors, namely movement intensity, movement tasks, and ball possession, which possibly influence the device’s concurrent validity.

## 2. Materials and Methods

Seven female professional basketball players (first division in Germany) took part in this cross-sectional validation study. All participants gave written informed consent to the experimental procedure, which was approved by the ethics committee of the University of Freiburg (429/18) and was in accordance with the latest revision of the Declaration of Helsinki.

### 2.1. Data Acquisition

Data from a single wearable tracking device, which included an IMU with a sampling frequency of 100 Hz (OptimEye S5, Catapult Sports, Melbourne, Australia), were compared to data from a 3D motion analysis (MA) system operating at 200 Hz (Vicon Motion Systems, Oxford, UK).

**Figure 1:**
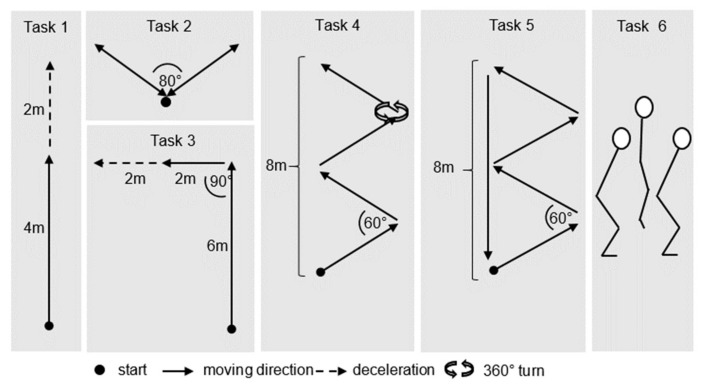
Graphic representation of performed movement tasks. Each task was performed at three intensities (low–moderate–high) both with and without ball possession. Adapted from Luteberget et al. [20], Task 1 consisted of a straight-line acceleration immediately followed by a deceleration to a stop. Task 2 consisted of two diagonal forward/backward movements. Task 3 was a straight-line acceleration followed by a 90° turn and deceleration to a stop. Task 4 was a zig-zag course including a 360° turn. The course was executed with forward movements and change of direction cuttings. For Task 5, five continuous laps of the same zig-zag course were executed without the 360° turn. Task 6 was added to expand the test battery through a single counter-movement jump to include motion in the vertical plane.

Data were collected during the performance of six team sport-specific movements adopted from Luteberget and colleagues [20] (Figure 1) after a standardized warm-up. All tasks were performed with and without ball handling. Under both conditions, participants performed each task (except for Task 6) at three intensity levels ranging from low, moderate, to high intensity, based on the athlete’s perceived effort. Task 6 was a maximum vertical jump performed at high intensity only. A total of 1120 trials were recorded. Twenty-seven trials had to be excluded from further analysis due to insufficient data quality. The resulting number of trials (1093) was sufficient for the power analysis calculated a priori based on previously-reported results by our working group [13].

Throughout the whole measurement process, participants wore the tracking device in a customized upper body harness provided by the vendor. The IMU was located in a customized pocket between the athlete’s shoulder blades. The same device was used for all participants to eliminate inter-device variability [21,22]. Three retro-reflective markers (∅ 14 mm) were attached directly to the device’s case to prevent displacement artefact between the markers and the device. To synchronize the measurement systems during post processing, participants performed a vertical jump serving as trigger prior to each trial, followed by 5 s of standing still before executing the task. After each task, participants stood still for 10 s to evaluate the convergence time for each sensor fusion algorithm.

### 2.2. Data Processing

Both the MA system data and the IMU raw data were exported to Microsoft Excel (Microsoft Excel 2013, Version 15.0, Redmond, WA, USA) using the respective manufacturer-supplied software (MA: Nexus 2, Vicon Motion Systems, Oxford, UK; IMU: Sprint 5.1.7, Catapult Sports, Melbourne, Australia). If not stated otherwise, all further calculations were performed using customized scripts (Matlab R2018a, MathWorks, Natick, MA, USA).

#### 2.2.1. Motion Analysis (MA) Data

The position of a single virtual marker was calculated at the estimated position of the IMU within the dimensions of the tracking device. A fourth order, zero-lag, low-pass digital Butterworth filter was applied to reduce noise from the MA positional data in the x-, y-, and z-axes. According to a residual analysis [23], a cut-off frequency of 6 Hz was chosen for all trials since the standard deviation (SD) of residuals between all trials was <1 Hz. The cut-off frequency was then corrected to 6.23 Hz [23] to exclude phase shift due to dual pass filtering. Acceleration values (m·s^−2^) in all three orthogonal planes were calculated through double numerical differentiation of the processed MA data. Magnitudes were calculated for the vectors of resultant 3D acceleration, resultant horizontal acceleration, and vertical acceleration according to the following formulas:

$$\begin{array}{l} \left|{\mathrm{acc}}_{\mathrm{res}}\right|=\sqrt{x^{2}+y^{2}+z^{2}}\\ \left|{\mathrm{acc}}_{\mathrm{hor}}\right|=\sqrt{x^{2}+y^{2}}\\ \left|{\mathrm{acc}}_{\mathrm{vert}}\right|=\sqrt{z^{2}} \end{array}$$

#### 2.2.2. Kalman Filter (KF)

The resultant acceleration vector (|acc_res_|) was provided by the manufacturer’s software (after KF-processing) with only one continuous variable: the combined acceleration of the x-, y-, and z-vectors corrected for gravity. This variable was previously validated under sport-specific conditions [14,24]. A low-pass digital filter (fourth order Butterworth) with a cut-off frequency of 8 Hz (9.97 Hz corrected) was applied for noise reduction. The cut-off frequency was chosen for all trials after residual analysis (SD < 1.0 Hz) and on the basis of the lowest mean bias compared to the MA system. Since the choice of variables was provided by the manufacturer, the data necessary to calculate |acc_hor_| and |acc_vert_| acceleration were not provided. Evaluation of KF validity is thus restricted to |acc_res_| only.

#### 2.2.3. Complementary Filter (CF)

A CF algorithm was manually implemented using routines written in C++ (Microsoft Visual C++ 2017, Redmond, WA, USA). The filter was originally developed to navigate unmanned aerial vehicles, but has since been successfully adapted to correct attitude during simulated sport-specific orientations [13,25]. Before running the CF, raw accelerometer and gyroscope data were converted to m·s^−2^ and low-pass filtered (fourth order Butterworth) to eliminate miscalculations by the CF algorithm. A cut-off frequency of 8 Hz (9.97 Hz corrected) was chosen after residual analysis for all trials (SD < 1.0 Hz). Based on previous studies that implemented CF algorithms for human motion analysis, an initial filtering gain of 0.0072 was chosen, which was adjusted throughout the calculations by an adaptive gain loop. Original accelerometer data in the x-, y-, and z-axes were rotated using the orientation quaternion calculated by the CF. The resulting coordinate system was defined by the z-axis running parallel to earth’s gravity vector. Gravitational influence was eliminated by subtracting 9.81 m·s^2^ from the resulting z-value. The x- and y-axes were not aligned to earth’s coordinate system since magnetometer data are disturbed by metal constructions in indoor facilities. Similar to the MA data, the magnitude of acceleration was calculated for |acc_res_|, |acc_hor_| and |acc_vert_|.

### 2.3. Data Analyses

Resulting CF, KF, and MA data were equally resampled from 100 Hz to 10 Hz and 5 Hz by continuously averaging every 10 or 20 data points, respectively. A fast Fourier transformation carried out on MA data showed the highest frequency components in the resulting power spectrum at 2 Hz, irrespective of the performed movement. The chosen temporal resolutions therefore are in accordance with the Nyquist–Shannon sampling theorem, which suggests that the minimum sampling frequency should be double the frequency of its highest frequency component [26].

CF, KF, and MA data were synchronized by cross-correlation against the trigger peak signal. The trigger signals and waiting phases prior to and post each task were excluded from further analysis. Based on typical applications of monitoring data for performance evaluations, mean and peak values of |acc_res_|^CF/MA/KF^, |acc_hor_|^CF/MA^, and |acc_vert_|^CF/MA^ were calculated. The resulting values were categorized into pre-defined acceleration bands. For mean values, acceleration bands were: 0–1 m·s^−2^, 1–2 m·s^−2^, 2–3 m·s^−2^, 3–4 m·s^−2^, 4–5 m·s^−2^, 5–6 m·s^−2^, and >6 m·s^−2^, and for peak values: 0–5 m·s^−2^, 5–10 m·s^−2^, 10–15 m·s^−2^, 15–20 m·s^−2^, 20–25 m·s^−2^, 25–30 m·s^−2^, and >30 m·s^−2^.

**Figure 2:**
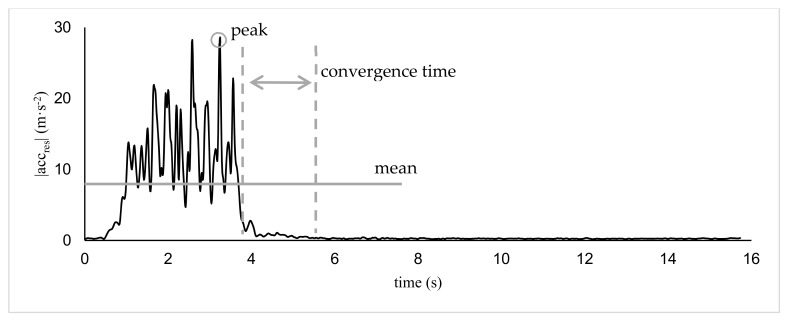
Inertial Measurement Unit (IMU) data analysis. Representative data of one trial in the 100 Hz temporal resolution is shown for |acc_res_| (Kalman Filter, KF).

Convergence time was calculated referring to baseline ranges for CF, MA, and KF, which were defined as the corridor between the upper and lower bounds of the mean ± 2SD during the last 3 s of the waiting phase after movement execution. The start of convergence was defined as the time point when the last value exceeded the baseline level +2.5 m·s^−2^ of the 100 Hz MA data. This threshold was chosen as this amount of acceleration was physically related to actual movements of the athlete rather than reflecting respiratory-induced torso movements. Further, values of 2.5 m·s^−2^ were also reached during lower intensities, which makes this threshold appropriate for all performed intensities of motion and inter-athlete variation in movement execution. Convergence time was then calculated as total time elapsed until the signal dropped back to the predefined baseline range (Figure 2).

### 2.4. Method Comparison

Following suggestions made by Atkinson and Nevill [27], the accordance between CF/KF and MA data was calculated for mean and peak acceleration magnitudes in relevant movement dimensions. Mean bias (MB), SD, 95% limits of agreement (LoA; 27), Spearman’s ρ (r_s_), coefficient of variation (CV; [28]), and root mean square error (RMSE) were calculated to determine the ability of the IMU to quantify mean and peak accelerations. MB ± SD, LoA, and RMSE provide information about the amount of error, while Spearman’s ρ and CV quantify the variance of the data. Bland–Altman plots were constructed to visually evaluate the agreement between IMU and MA data [29].

Statistical analyses were performed using JMP Version 13.1.0 (SAS Institute Inc., Cary, NC, USA). Shapiro–Wilk tests showed homogeneous data for convergence time. A one-way ANOVA was calculated between groups to determine differences in convergence time between CF, KF, and MA. Two-sided *t*-tests were calculated post-hoc to determine differences in the mean between single groups. The Bonferroni-corrected adjusted alpha level was set to α = 0.017.

## 3. Results

### 3.1. Internal Factors

**Figure 3:**
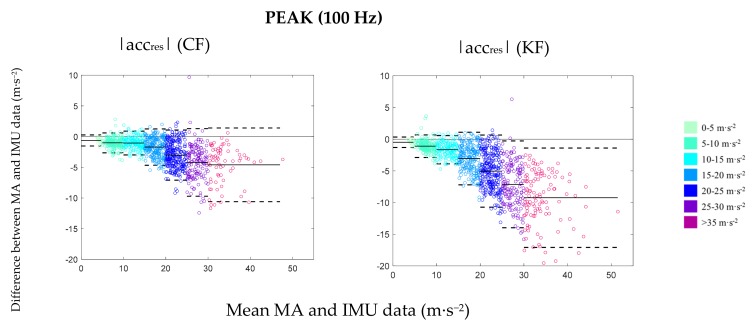
Bland–Altman plots showing the relationship between motion analysis (MA) and IMU data using Complementary Filter (CF) and KF for peak values at the 100 Hz temporal resolution. Mean and peak values for each trial were categorized according to pre-defined bands. For each acceleration band, 95% limits of agreement (LoA) (dashed line) and mean bias (solid line) are displayed with reference to zero (black solid line).

At the original resolution of 100 Hz, both filtering algorithms tended to overestimate MA acceleration values over all trials and dimensions by 5.67% to 26.10%. Generally, CF showed lower errors and higher accuracy for mean and peak values across all temporal resolutions. For KF measurement, outcomes indicate large ranges for LoA of ≥1 m·s^−2^ for mean and ≥8 m·s^−2^ for peak values. MB data further shows substantial overestimation by KF (MB_mean_ = 13.93%–77.21%; MB_peak_ = 22.24%–37.17%) over all trials and temporal resolutions. Differences between both filtering algorithms were most prominent for higher intensities (Figure 3). Outcome measures of agreement comparing both sensor fusion methods are shown in Table 1.

In general, lower temporal resolutions were associated with higher accuracy of the CF data than higher temporal resolutions (Table 1). The 5 Hz resolution demonstrated the best relationship between the CF and MA system data. In contrast, errors increased with lower temporal resolutions for the KF results. The results are presented for |acc_res_| and are similar to |acc_hor_| and |acc_vert_|.

**Figure 4:**
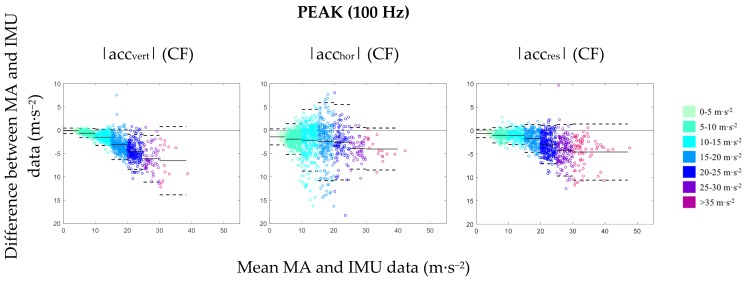
Bland–Altman plots showing the relationship between MA system data and CF data for peak values of |acc_res_|, |acc_hor_|, and |acc_vert_|. Magnitude values for each trial were categorized according to pre-defined acceleration bands. For each acceleration band, 95% LoA (dashed line) and mean bias (solid line) are displayed with reference to zero (black solid line).

Comparing the values measured by the IMU, the lowest agreement was found for |acc_hor_| in terms of LoA, which ranged from 1.00–1.08 m·s^−2^ for mean and 7.57–9.45 m·s^−2^ for peak variables regardless of temporal resolution, acceleration band, or performed movement task (Figure 4). The highest agreement was observed for |acc_vert_| variables across all conditions, while |acc_res_| showed a moderate relationship (Table 1).

### 3.2. External Factors

Across all conditions, the relationship between IMU and MA system data decreased as the acceleration magnitude increased. Bland–Altman plots (Figure 3) clearly show a lower agreement between IMU and MA system data for acceleration magnitudes above 4.0 m·s^−2^ for mean values and 15.0 m·s^−2^ for peak values. For mean values, CF was able to maintain a comparable level of error over all intensities, while a moderate increase of MB and LoA in the higher acceleration bands was seen for peak values. Larger deviation and linear increases in error was observed in KF for mean and peak values as the acceleration magnitude increased. The results are presented for |acc_res_| and are in accordance with analysis of |acc_hor_| and |acc_vert_|. 

The mean convergence time over all trials was 3.81 ± 2.67 s for MA system data, 4.25 ± 2.58 s for CF data, and 4.34 ± 2.79 s for KF data. Accordingly, CF needed 0.44 s and KF 0.53 s longer than the MA reference signal to reach baseline level, a significant difference (CF: *p* = 0.0002, CI = −0.67 to −0.21; Cohen’s d = 0.167; KF: *p* = 0.0001, CI = −0.76 to −0.29, Cohen’s d = 0.19). However, no significant differences were observed between CF and KF (*p* = 0.4519, CI = −0.33 to 0.14, Cohen’s d = 0.33). 

No substantial differences were observed between tasks 1 to 5 independent of the variable, applied sensor fusion, temporal resolution, or intensity (Table 2). In contrast, error and variance in Task 6 for both mean and peak variables was about twice as large as other tasks. No differences were seen in the amount of error and variance with respect to ball possession.

## 4. Discussions

This study aimed to evaluate the concurrent validity of acceleration magnitudes derived from a commercially-available tracking device after different techniques of data processing. A 3D MA system served as the criterion measure during team sport-specific movements. With respect to internal factors, our results showed superior results for CF compared to KF across all conditions. Furthermore, findings of this study indicate an impaired concurrent validity of the IMU at higher temporal resolutions, for both horizontal axes as well as for higher movement intensities. 

### 4.1. Internal Factors

When working with IMU-based tracking devices, the technical properties of the hardware require a significant amount of data processing. High discrepancies between accelerometer data and reference measures during sporting activities have been demonstrated, usually due to insufficient sensor fusion due to missing gravity compensation [13,30,31]. Our results demonstrate that the type of sensor fusion itself influences the tracking device’s concurrent validity. In direct comparison to the manufacturer’s KF, the CF clearly showed lower errors against the MA reference data across all conditions. The exact reason for the algorithms’ divergent behavior remains unclear since the exact functions of KF are confidential. Advantages for the CF are its faster convergence as changes in activity likely include a brief stop of motion. Those activities have been reported to occur every 2–4 s in multidirectional team sports, adding 500–3000 changes of activity over a game [32]. Ricci and colleagues [6] demonstrated an immediate rate of convergence for a self-implemented CF algorithm during stop-after-motion trials performed by a robotic arm. In comparison, KF required about 10 s to reach a stable signal after stopping [6]. Even though our study yielded no significant differences, absolute values demonstrated slight differences in convergence time of about 0.1 s between both filters with lower values for the CF. Considering the fast-paced nature of typical team sport movements, such as quick changes of direction, this could easily explain differences in validity. A fast convergence behavior might be preferable for tracking team sport athletes, as it enables the algorithm to reflect the intermittent nature of these sports by following sudden changes in acceleration.

According to our results, the validity of the tracking device further depends on the extent of resampling. For the CF, only marginal differences were seen when resampling the 100 Hz data to 10 Hz. However, lower errors indicating a better accuracy were observed for 5 Hz. This is probably caused by lower overall acceleration amplitudes, possibly resulting from the averaging process. The resampling by averages was chosen because the original sampling frequency of 100 Hz was likely to cause oversampling of the true acceleration, and therefore inaccurately measuring unintentional movements of the sensor within the harness due to external impacts rather than actual body motion. A substantial error between peak vertical acceleration of the thoracic segment between IMU and MA data has been observed in a similar experiment [33]. The authors explained this discrepancy through artefacts caused by the elastic material of the harness. The process of resampling, as performed in our study, might have reduced this specific source of noise in the data. Resampling might ensure higher congruence between IMU measures and actual upper body motion. In addition to yielding higher validity, resampling to 5 Hz might be more appropriate to reflect actual movements of the upper body. This should be considered in future studies evaluating the criterion validity of IMUs. Regardless of the temporal resolution, validity was substantially impaired with respect to horizontal acceleration. Thus, providing meaningful information in this movement plane is difficult, especially at higher intensities. The strongest relationships were found for the vertical magnitude. The accuracy of the resultant acceleration lies within a range comparable to previous validation studies [14,19]. Similar observations were made during a highly controlled technical validation of a tracking device equivalent to the one used in this study [22]. Higher percentage errors between the device and an industrial IMU were found on the x- and y-axis compared to the z-axis during steadily oscillating movements. Further, larger errors in the horizontal plane were reported for IMU-based variables compared between multiple devices [22,24]. The authors assigned the reduced accuracy of both horizontal axes to a possible error in device calibration. Inconsistencies of the specified measurement ranges in each axis could further explain this result. Gravitational acceleration primarily acts on the z-axis, thus increasing raw data by 9.81 m·s^−2^ compared to the x- and y-axes, which in itself could require adjustments to axis-specific measurement ranges. 

### 4.2. External Factors

The second aim of this study was to determine how factors of sporting activities such as movement intensity, movement task, and ball possession could impair the device’s validity. Again, convergence might play a role and might also explain the stronger discrepancy between IMU and MA at higher intensity levels. In lower acceleration bands, both sensor fusion algorithms demonstrated a comparable level of accuracy. However, they drifted apart after 15.0 m·s^−2^ at peak values and 4.0 m·s^−2^ at mean values. Therefore, KF data well exceeds acceptable limits of agreement and we conclude that it is not valid at these intensity ranges. Comparable findings were shown during treadmill locomotion, with IMUs overestimating acceleration values by more than 1.5 g at higher acceleration magnitudes [19]. While the authors suggested the relatively loose fit of the harness as the source of error, our results suggest that the delayed convergence of sensor fusion hinders an exact measurement of the true signal in higher intensities and also affects the device’s accuracy. Researchers and practitioners should be aware of the increased error at higher intensities and interpret these data with caution. However, choosing a tightly-fitting harness and an appropriate data processing method could help to decrease errors to an acceptable level.

In contrast to movement intensity, there was little evidence to suggest that the performed movement itself affected the IMU’s validity. No differences in accuracy were observed during straight-line and change-of-direction movements, although the IMU tended to overestimate true acceleration for the vertical jump. While the former tasks consisted of complex movements, including changes of directions, multidirectional acceleration, and deceleration, the latter was intended to reflect a single vertical jump. Considering that the primary movement direction of jumping is in the vertical axis, higher accuracies could be expected due to the IMU’s axis specificity. However, during a maximum vertical jump, higher absolute accelerations were reached, which seemed to have a stronger influence on the device’s validity. In contrast to our findings, Wundersitz and colleagues [14] did not find any explicit differences between a double legged jump and other sport-related movement patterns performed within a team sport-specific circuit. However, as the jump was not performed as an isolated task, lower absolute values than our study can be expected. Instead, a tackling motion performed at the end of the circuit seems to be more comparable to our jumping task as both movements represent a single short bout of maximum intensity. The results of both studies indicate that IMUs can be applied to quantify complex movement tasks but have difficulties when accurately measuring rapid movements (<1 s) performed at highest intensities.

Team sports not only have specific movement patterns, but also require a certain amount of ball handling using either the lower or upper limbs. Indoor sports such as basketball, handball, or volleyball are typically restricted to handed ball contact, which likely induces a certain amount of acceleration due to a transfer of arm motion to the upper body. This might affect the device’s concurrent validity. However, according to our results, accuracy was not impaired by ball handling. It should be noted that this study did not evaluate the ability of the IMU to correctly measure upper body motion. In an extensive experiment, a tri-axial accelerometer did not serve as a valid representation of the thoracic segment with respect to vertical acceleration [33]. An exact alignment between local and global coordinate systems has not been possible. Thus, the validity of tracking devices remains inconclusive. Further research should focus on the validity of upper body-mounted devices and consider possible influences such as ball handling.

### 4.3. Limitations

As a limitation of this study, the proposed CF algorithm was only able to correct the tracking device’s attitude with respect to the global coordinate system and could not calculate the device’s heading. This hinders the alignment between local and global x- and y-axes and forces the calculation of horizontal movement proportions by the resultant horizontal acceleration. The respective x- and y-axes cannot be quantified. The direction of movement remains unclear and thereby hinders discrimination of acceleration and deceleration on the horizontal axes. In outdoor environments, magnetometer data can be used to estimate the devices’ heading, however, when indoors, metal or electric constructions induce ferromagnetic disturbances that distort magnetometer readings. Even though intense research in indoor navigation has highlighted this problem, it remains unresolved. Promising approaches include creating specific magnetic maps and/or landmarks to determine a device’s absolute position and orientation [34,35]. However, these techniques have not yet been tested in sport-specific settings.

As the MA system records displacement data rather than acceleration itself, a certain amount of high-frequency noise must be accounted for during numerical differentiation of positional MA data. Even though appropriate filtering algorithms partially dampen these errors, an influence on the criterion data cannot be excluded. Still, MA system data serve as an accepted criterion for validation of acceleration estimates in player monitoring systems [14,36]. 

## 5. Conclusions

Given the results of this study, wearable tracking devices containing an IMU can be used for indoor player monitoring with acceptable validity to determine magnitudes of acceleration during team sport-specific movements. Researchers and practitioners should be well aware of internal and external factors affecting the device’s validity. In particular, the choice of sensor fusion possibly influenced the IMU’s accuracy. Overall, the implemented CF algorithm showed superior accuracy over the manufacturer-dependent KF due to its faster convergence. According to our results, a resampled 5 Hz resolution showed the strongest relationship between CF and MA data, but still met the requirements of Nyquist–Shannon’s sampling theorem. Caution must be given to the impaired accuracy in the horizontal plane. While the CF data in the 5 Hz resolution can be seen as valid, KF accuracy should be seen critically, especially for higher intensities. Regarding external factors, movement intensity showed the highest impact on device accuracy with substantially larger errors found for higher acceleration bands. In contrast, movement task and ball possession did not considerably affect the device’s validity. For researchers and practitioners to rely on the device’s data, they should be aware of the implemented data processing methods as well as the underlying hardware.

Overall, our results legitimize the use of IMUs for accurate measurement of players’ workloads in team sports under the premise of adequate data processing. The manufacturer-independent implementation of a CF with a resampled temporal resolution of 5 Hz is recommended since it enables the accurate quantification of movement in vertical and three-dimensional acceleration efforts. Further, this study proposes and validates an approach of raw data treatment, which allows controlled and transparent processing, long-term data collection, and avoids inaccuracies due to software updates. This broadens the content of information given in acceleration-based activity profiles and leads to a deeper understanding of true physical demands in indoor team sports.

## Figures and Tables

**Table 1:** Analysis of agreement between CF or KF data and MA system data over all trials (*n* = 1093). Outcomes for mean and peak values are presented at each temporal resolution. Results refer to |acc_res_|, |acc_hor_| and |acc_vert_|. MB, mean bias; SD, standard deviation; 95% LoA, 95% limits of agreement; CI, confidence intervals; RMSE, root mean square error; r_s_, Spearman’s correlation coefficient; CV, coefficient of variation.

		MEAN					PEAK				
		MB ± SD (m·s^−2^)	95% LoA ± CI (m·s^−2^)	r_s_	CV (%)	RMSE (m·s^−2^)	MB ± SD (m·s^−2^)	95% LoA ± CI (m·s^−2^)	r_s_	CV (%)	RMSE (m·s^−2^)
**100**	|acc_vert_|(CF)	−0.16 ± 0.16	−0.48 to 0.16 ± 0.02	0.99	5.40	0.23	−2.63 ± 2.27	−7.07 to 1.81 ± 0.23	0.98	7.85	3.57
	|acc_hor_|(CF)	−0.38 ± 0.32	−1.01 to 0.25 ± 0.03	0.95	19.44	0.49	−2.24 ± 3.16	−8.44 to 3.96 ± 0.32	0.87	28.62	3.87
	|acc_res_|(CF)	−0.42 ± 0.31	−1.02 to 0.18 ± 0.03	0.99	7.34	0.52	−2.31 ± 2.25	−6.71 to 2.10 ± 0.23	0.97	10.05	3.30
	|acc_res_|(KF)	−0.57 ± 0.41	−1.38 to 0.25 ± 0.04	0.99	5.99	0.70	−4.18 ± 3.68	−11.39 to 3.03 ± 0.38	0.95	12.10	5.57
**10 Hz**	|acc_vert_|(CF)	−0.14 ± 0.16	−0.47 to 0.18 ± 0.02	0.99	5.54	0.22	−2.15 ± 2.28	−6.63 to 2.32 ± 0.23	0.97	9.23	3.14
	|acc_hor_|(CF)	−0.37 ± 0.32	−0.99 to 0.25 ± 0.03	0.95	20.11	0.49	−2.04 ± 3.11	−8.12 to 4.05 ± 0.32	0.86	30.13	3.71
	|acc_res_|(CF)	−0.40 ± 0.29	−0.98 to 0.17 ± 0.03	0.98	7.46	0.50	−1.87 ± 2.12	−6.03 to 2.29 ± 0.22	0.97	10.63	2.83
	|acc_res_|(KF)	−0.66 ± 0.55	−1.75 to 0.43 ± 0.06	0.98	8.90	0.86	−3.85 ± 3.52	−10.74 to 3.05 ± 0.36	0.94	12.74	5.21
**5 Hz**	|acc_vert_|(CF)	−0.12 ± 0.15	−0.40 to 0.17 ± 0.02	0.97	10.57	0.19	−0.34 ± 0.84	−1.99 to 1.32 ± 0.09	0.95	12.08	0.91
	|acc_hor_|(CF)	−0.32 ± 0.32	−0.95 to 0.30 ± 0.03	0.91	29.13	0.46	−0.89 ± 2.67	−6.13 to 4.35 ± 0.27	0.75	43.21	2.81
	|acc_res_|(CF)	−0.33 ± 0.29	−0.91 to 0.24 ± 0.03	0.96	10.78	0.44	−0.14 ± 1.40	−2.88 to 2.60 ± 0.14	0.95	15.98	1.40
	|acc_res_|(KF)	−2.00 ± 1.23	−4.41 to 0.40 ± 0.13	0.90	16.48	2.35	−3.25 ± 2.85	−8.83 to 2.33 ± 0.29	0.85	22.56	4.32

**Table 2:** Analysis of agreement between CF data and MA system data for each movement task. Outcomes for mean and peak values are presented at a temporal resolution of 5 Hz and refer to |acc_res_| only. MB, mean bias; SD, standard deviation; 95% LoA, 95% limits of agreement; CI, confidence intervals; RMSE, root mean square error; r_s_, Spearman’s correlation coefficient; CV, coefficient of variation.

	|acc_res_| (CF, 5 Hz)				
	MB ± SD (m·s^−2^)	95% LoA ± CI (m·s^−2^)	r_s_	CV (%)	RMSE (m·s^−2^)
**MEAN**					
Task1	−0.27 ± 0.17	−0.61 to 0.07 ± 0.04	0.95	8.86	0.32
Task2	−0.39 ± 0.30	−0.98 to 0.20 ± 0.07	0.93	8.90	0.49
Task3	−0.31 ± 0.21	−0.72 to 0.10 ± 0.05	0.95	8.58	0.38
Task4	−0.35 ± 0.26	−0.85 to 0.16 ± 0.06	0.95	7.25	0.43
Task5	−0.22 ± 0.31	−0.83 to 0.40 ± 0.07	0.95	5.86	0.38
Task6	−0.73 ± 0.41	−1.53 to 0.06 ± 0.17	0.76	12.80	0.84
**PEAK**					
Task1	−0.50 ± 1.14	−2.74 to 1.74 ± 0.28	0.90	17.16	1.25
Task2	−0.58 ± 1.41	−3.35 to 2.19 ± 0.33	0.91	16.68	1.53
Task3	0.04 ± 1.14	−2.20 to 2.28 ± 0.27	0.95	15.32	1.14
Task4	0.47 ± 1.12	−1.73 to 2.67 ± 0.27	0.96	11.41	1.21
Task5	0.39 ± 1.14	−1.85 to 2.63 ± 0.27	0.96	11.39	1.20
Task6	−1.74 ± 2.06	−5.79 to 2.31 ± 0.84	0.61	10.42	2.69

## Supplementary Materials

Supplementary File 1
