# Supplementary material for: Validation of Wearable Sensors during Team Sport-Specific Movements in Indoor Environments
Source: Sensors (Basel). 2019 Aug 7;19(16):3458. doi: 10.3390/s19163458 (PMC6720677; doi:10.3390/s19163458)
Supplement: Supplementary File 1 [file sensors-19-03458-s001.zip › Table S1.docx]

|  |  | MB ± SD | LoA ± CI | r_s_ | CV (%) | RMSE (m·s^-2)^ |
| --- | --- | --- | --- | --- | --- | --- |
| \|acc_vert_\| | | | | | | |
| OVERALL (all trials included) | | | | | | |
| mean | all trials | -0.16 ± 0.16 | -0.48 to 0.16 0.02 | 0.99 | 5.40 | 0.23 |
| peak | all trials | -2.63 ± 2.27 | -7.07 to 1.81 ± 0.23 | 0.98 | 7.85 | 3.57 |
| BALL POSSESSION (all trials included, separated according to ball handling) | | | | | | |
| mean | without ball | -0.16 ± 0.17 | -0.49 to 0.18 ± 0.02 | 0.99 | 5.62 | 0.23 |
|  | with ball | -0.16 ± 0.16 | -0.47 to 0.15 ± 0.02 | 0.99 | 5.19 | 0.22 |
| peak | without ball | -2.67 ± 2.38 | -7.34 to 1.99 ± 0.35 | 0.97 | 8.28 | 3.58 |
|  | with ball | -2.58 ± 2.15 | -6.80 to 1.63 ± 0.31 | 0.98 | 7.44 | 3.36 |
| INTENSITY ( all trials included, separated according to acceleration band) | | | | | | |
| mean | 0-1 | -0.11 ± 0.03 | -0.17 to -0.06 ± 0.02 | 0.95 | 2.99 | 0.12 |
|  | 1-2 | -0.15 ± 0.16 | -0.47 to 0.17 ± 0.02 | 0.99 | 5.51 | 0.22 |
|  | 2-3 | -0.19 ± 0.18 | -0.54 to 0.16 ± 0.02 | 0.98 | 5.50 | 0.26 |
|  | 3-4 | -0.22 ±0.17 | -0.56 to 0.11 ± 0.03 | 0.97 | 4.24 | 0.28 |
|  | 4-5 | -0.25 ± 0.15 | -0.53 to 0.03 ± 0.03 | 0.98 | 2.54 | 0.29 |
|  | 5-6 | -0.28 ± 0.14 | -0.55 to -0.01 ± 0.04 | 0.96 | 2.17 | 0.31 |
|  | >6 | -0.31 ± 0.11 | -0.53 to -0.08 ± 0.04 | 0.91 | 1.82 | 0.33 |
| peak | 0-5 | -0.18 ± 0.32 | -0.80 to 0.45 ± 0.17 | 0.84 | 5.89 | 0.36 |
|  | 5-10 | -0.68 ± 0.53 | -1.73 to 0.36 ± 0.12 | 0.96 | 5.23 | 0.87 |
|  | 10-15 | -1.97 ± 1.22 | -4.36 to 0.42 ± 0.24 | 0.83 | 5.82 | 2.32 |
|  | 15-20 | -3.87 ± 1.91 | -7.62 to -0.12 ± 0.34 | 0.71 | 6.28 | 4.31 |
|  | 20-25 | -4.88 ± 2.93 | -10.63 to 0.86 ± 0.92 | 0.49 | 5.15 | 5.69 |
|  | 25-30 | -4.55 ± 2.84 | -10.11 to 1.01 ± 1.85 | 0.33 | 5.17 | 5.34 |
|  | >30 | -4.70 ± 3.14 | -10.85 to 1.45 ± 5.33 | 1.00 | 2.39 | 5.43 |
| TASK (trials categorized according to performed movement task, all intensities included) | | | | | | |
| mean | Task 1 | -0.15 ± 0.15 | -0.44 to 0.13 ± 0.04 | 0.98 | 5.48 | 0.21 |
|  | Task 2 | -0.14 ± 0.09 | -0.32 to 0.04 ± 0.02 | 0.99 | 3.61 | 0.17 |
|  | Task 3 | -0.17 ± 0.16 | -0.49 to 0.15 ± 0.04 | 0.99 | 5.39 | 0.23 |
|  | Task 4 | -0.19 ± 0.19 | -0.56 to 0.18 ± 0.04 | 0.99 | 5.15 | 0.27 |
|  | Task 5 | -0.17 ± 0.19 | -0.54 to 0.20 ± 0.04 | 0.99 | 2.48 | 0.25 |
|  | Task 6 | -0.05 ± 0.17 | -0.39 to 0.28 ± 0.07 | 0.90 | 8.26 | 0.18 |
| peak | Task 1 | -2.77 ± 2.86 | -8.38 to 2.84 ± 0.69 | 0.98 | 8.39 | 3.98 |
|  | Task 2 | -1.72 ± 1.47 | -4.59 to 1.16 ± 0.35 | 0.97 | 6.51 | 2.25 |
|  | Task 3 | -2.39 ± 2.10 | -6.50 to 1.72 ± 0.50 | 0.97 | 7.13 | 3.17 |
|  | Task 4 | -2.76 ± 2.10 | -6.88 to 1.36 ± 0.50 | 0.98 | 7.04 | 3.47 |
|  | Task 5 | -3.35 ± 2.12 | -7.50 to 0.81 ± 0.50 | 0.97 | 6.69 | 3.96 |
|  | Task 6 | -3.09 ± 2.71 | -8.39 to 2.21 ± 1.11 | 0.78 | 11.07 | 4.09 |
| \|acc_hor_\| | | | | | | |
| OVERALL (all trials included) | | | | | | |
| mean | all trials | -0.38 ± 0.32 | -1.01 to 0.25 ± 0.03 | 0.95 | 19.44 | 0.49 |
| peak | All trials | -2.24 ± 3.16 | -8.44 to 3.96 ± 0.32 | 0.87 | 28.62 | 3.87 |
| BALL POSSESSION (all trials included, separated according to ball handling) | | | | | | |
| mean | without ball | -0.36 ± 0.36 | -1.06 to 0.34 ± 0.05 | 0.93 | 21.47 | 0.50 |
|  | with ball | -0.39 ± 0.28 | -0.95 to 0.16 ± 0.04 | 0.97 | 16.99 | 0.48 |
| peak | without ball | -2.09 ± 3.29 | -8.54 to 4.36 ± 0.48 | 0.88 | 29.19 | 3.90 |
|  | with ball | -2.38 ± 3.03 | -8.32 to 3.56 ± 0.44 | 0.86 | 28.09 | 3.85 |
| INTENSITY ( all trials included, separated according to acceleration band) | | | | | | |
| mean | 0-1 | -0.50 ± 0.45 | -1.39 to 0.39 ± 0.15 | -0.05 | 22.32 | 0.67 |
|  | 1-2 | -0.36 ± 0.30 | -0.95 to 0.23 ± 0.03 | 0.96 | 11.91 | 0.47 |
|  | 2-3 | -0.42 ± 0.33 | -1.07 to 0.23 ± 0.05 | 0.94 | 8.71 | 0.54 |
|  | 3-4 | -0.50 ± 0.44 | -1.36 to 0.35 ±0.11 | 0.85 | 7.49 | 0.66 |
|  | 4-5 | -0.74 ± 0.48 | -1.69 to 0.20 ± 0.21 | 0.86 | 3.69 | 0.88 |
|  | 5-6 | -1.33 ± 0.30 | -1.92 to -0.74 ±0.28 | 0.78 | 2.66 | 1.36 |
|  | >6 |  |  |  |  |  |
| peak | 0-5 | -2.61 ± 2.44 | -7.40 to 2.18 ± 0.89 | 0.64 | 18.03 | 3.57 |
|  | 5-10 | -2.63 ± 2.72 | -7.96 to 2.70 ± 0.46 | 0.53 | 19.71 | 3.78 |
|  | 10-15 | -2.23 ± 3.59 | -9.28 to 4.81 ± 0.72 | 0.42 | 11.37 | 4.23 |
|  | 15-20 | -1.53 ± 2.99 | -7.39 to 4.33 ± 0.75 | 0.39 | 7.44 | 3.35 |
|  | 20-25 | -1.22 ± 3.78 | -8.63 to 6.19 ±1.332 | 0.43 | 6.19 | 3.95 |
|  | 25-30 | -3.09 ± 2.53 | -8.05 to 1.87 ± 1.43 | 0.32 | 4.93 | 3.97 |
|  | >30 | -2.73 ± 5.56 | -13.63 to 8.18 ± 5.24 | 0.82 | 6.78 | 6.00 |
| TASK (trials categorized according to performed movement task, all intensities included) | | | | | | |
| mean | Task 1 | -0.26 ± 0.19 | -0.63 to 0.10 ± 0.05 | 0.95 | 11.49 | 0.32 |
|  | Task 2 | -0.40 ± 0.29 | -0.97 to 0.17 ± 0.07 | 0.97 | 7.96 | 0.50 |
|  | Task 3 | -0.31 ± 0.18 | -0.66 to 0.03 ± 0.04 | 0.98 | 7.35 | 0.36 |
|  | Task 4 | -0.37 ± 0.23 | -0.83 to 0.09 ± 0.06 | 0.98 | 6.33 | 0.44 |
|  | Task 5 | -0.34 ± 0.39 | -1.11 to 0.43 ± 0.09 | 0.99 | 7.22 | 0.52 |
|  | Task 6 | -0.93 ± 0.45 | -1.81 to -0.05 ± 0.18 | 0.71 | 19.89 | 1.03 |
| peak | Task 1 | -3.19 ± 3.09 | -9.24 to 2.86 ± 0.74 | 0.89 | 26.71 | 4.43 |
|  | Task 2 | -2.28 ± 2.06 | -6.33 to 1.76 ± 0.49 | 0.94 | 16.92 | 3.08 |
|  | Task 3 | -1.75 ± 2.33 | -6.31 to 2.81 ± 0.55 | 0.95 | 20.03 | 2.91 |
|  | Task 4 | -0.84 ± 2.60 | -5.94 to 4.26 ± 0.62 | 0.92 | 18.75 | 2.73 |
|  | Task 5 | -1.13 ± 2.34 | -5.72 to 3.46 ± 0.55 | 0.96 | 14.82 | 2.60 |
|  | Task 6 | -8.25 ± 4.13 | -16.36 to -0.15 ± 1.69 | 0.52 | 38.22 | 9.22 |
| \|acc_res_\| (CF) | | | | | | |
| OVERALL (all trials included) | | | | | | |
| mean | all trials | -0.42 ± 0.31 | -1.02 to 0.18 ± 0.03 | 0.99 | 7.34 | 0.52 |
| peak | All trials | -2.31 ± 2.25 | -6,71 to 2.10 ± 0.23 | 0.97 | 10.05 | 3.30 |
| BALL POSSESSION (all trials included, separated according to ball handling) | | | | | | |
| mean | Without ball | -0.39 ± 0.33 | -1.04 to 0.25 ± 0.05 | 0.99 | 7.85 | 0.51 |
|  | with ball | -0.44 ± 0.28 | -0.99 to 0.11 ± 0.04 | 0.99 | 6.69 | 0.52 |
| peak | Without ball | -2.36 ±2.31 | -6.89 to 2.17 ± 0.34 | 0.97 | 10.07 | 3.30 |
|  | with ball | -2.25 ± 2.19 | -6.53 to 2.03 ±0.31 | 0.97 | 10.01 | 3.14 |
| INTENSITY ( all trials included, separated according to acceleration band) | | | | | | |
| mean | 0-1 |  |  |  |  |  |
|  | 1-2 | -0.40 ± 0.29 | -0.96 to 0.16 ± 0.03 | 0.98 | 7.51 | 0.49 |
|  | 2-3 | -0.45 ± 0.31 | -1.06 to 0.17 ± 0.03 | 0.98 | 7.33 | 0.54 |
|  | 3-4 | -0.48 ± 0.31 | -1.09 to 0.13 ± 0.04 | 0.98 | 5.84 | 0.57 |
|  | 4-5 | -0.53 ± 0.34 | -1.20 ± 0.13 to 0.06 | 0.96 | 5.71 | 0.63 |
|  | 5-6 | -0.55 ± 0.38 | -1.28 to 0.19 ± 0.09 | 0.97 | 5.16 | 0.66 |
|  | >6 | -0.55 ± 0.39 | -1.32 to 0.21 ± 0.11 | 0.96 | 4.08 | 0.68 |
| peak | 0-5 | -0.73 ± 0.47 | -1.66 to 0.19 ± 0.33 | 0.73 | 8.10 | 0.86 |
|  | 5-10 | -1.05 ± 0.84 | -2.70 to 0.60 ± 0.20 | 0.82 | 10.51 | 1.34 |
|  | 10-15 | -1.24 ± 1.11 | -3.42 to 0.95 ±0.29 | 0.84 | 6.48 | 1.66 |
|  | 15-20 | -2.48 ± 1.97 | -6.34 to 1.39 ± 0.39 | 0.69 | 6.15 | 3.16 |
|  | 20-25 | -3.34 ± 2.73 | -8.68 to 2.01 ± 0.61 | 0.54 | 5.35 | 4.31 |
|  | 25-30 | -3.82 ± 2.66 | -9.04 to 1.39 ± 0.88 | 0.40 | 4.54 | 4.65 |
|  | >30 | -3.05 ± 2.98 | -8.90 to 2.81 ± 1.49 | 0.77 | 6.52 | 4.24 |
| TASK (trials categorized according to performed movement task, all intensities included) | | | | | | |
| mean | Task 1 | -0.30 ± 0.20 | -0.69 to 0.09 ±0.05 | 0.99 | 5.41 | 0.36 |
|  | Task 2 | -0.46 ± 0.29 | -1.02 to 0.11 ± 0.07 | 0.97 | 6.02 | 0.54 |
|  | Task 3 | -0.37 ± 0.23 | -0.81 to 0.08 ± 0.05 | 0.99 | 4.72 | 0.43 |
|  | Task 4 | -0.44 ± 0.30 | -1.02 to 0.14 ± 0.07 | 0.98 | 4.90 | 0.53 |
|  | Task 5 | -0.40 ± 0.35 | -1.09 to 0.29 ± 0.08 | 0.99 | 3.45 | 0.53 |
|  | Task 6 | -0.75 ± 0.42 | -1.58 to 0.07 ± 0.17 | 0.85 | 10.33 | 0.86 |
| peak | Task 1 | -3.30 ± 2.66 | -8.52 to 1.92 ± 0.64 | 0.98 | 8.88 | 4.24 |
|  | Task 2 | -1.61 ± 1.90 | -5.34 to 2.12 ± 0.45 | 0.96 | 10.11 | 2.49 |
|  | Task 3 | -2.13 ± 2.07 | -6.18 to 1.92 ± 0.49 | 0.97 | 9.95 | 2.96 |
|  | Task 4 | -1.93 ± 2.06 | -5.96 to 2.10 ± 0.49 | 0.96 | 9.53 | 2.82 |
|  | Task 5 | -2.08 ± 1.90 | -5.80 to 1.65 ± 0.45 | 0.98 | 7.91 | 2.81 |
|  | Task 6 | -3.81 ± 2.36 | -8.43 to 0.80 ± 0.96 | 0.84 | 9.30 | 4.47 |
| \|acc_res_\| (KF) | | | | | | |
| OVERALL (all trials included) | | | | | | |
| mean | all trials | -0.57 ± 0.41 | -1.38 to 0.25 ±0.04 | 0.99 | 5.99 | 0.70 |
| peak | All trials | -4.18 ± 3.68 | -11.39 to 3.03 ± 0.38 | 0.95 | 12.10 | 5.57 |
| BALL POSSESSION (all trials included, separated according to ball handling) | | | | | | |
| mean | with ball | -0.58 ± 0.46 | -1.48 to 0.31 ± 0.07 | 0.99 | 6.55 | 0.74 |
|  | without ball | -0.55 ±0.37 | -1.28 to 0.17 ±0.05 | 0.99 | 5.36 | 0.67 |
| peak | With ball | -4.44 ± 3.83 | -11.96 to 3.07 ±0.56 | 0.95 | 11.48 | 5.87 |
|  | Without ball | -3.93 ± 3.51 | -10.80 to 2.95 ± 0.50 | 0.96 | 12.51 | 5.26 |
| INTENSITY ( all trials included, separated according to acceleration band) | | | | | | |
| mean | 0-1 |  |  |  |  |  |
|  | 1-2 | -0.49 ± 0.35 | -1.18 to 0.19 ±0.04 | 0.98 | 5.86 | 0.60 |
|  | 2-3 | -0.62 ± 0.41 | -1.43 to 0.19 ± 0.05 | 0.99 | 6.20 | 0.75 |
|  | 3-4 | -0.74 ± 0.41 | -1.54 to 0.07 ± 0.05 | 0.97 | 6.42 | 0.84 |
|  | 4-5 | -0.86 ± 0.43 | -1.70 to -0.02 ± 0.07 | 0.95 | 6.23 | 0.96 |
|  | 5-6 | -0.98 ± 0.46 | -1.88 tp -0.07 ±0.11 | 0.96 | 5.52 | 1.08 |
|  | >6 | -1.06 ± 0.47 | -1.97 to -0.14 ±0.13 | 0.95 | 4.35 | 1.15 |
| peak | 0-5 | -0.60 ± 0.48 | -1.54 to 0.35 0.34 | 0.81 | 6.66 | 0.76 |
|  | 5-10 | -1.24 ±0.99 | -3.18 to 0.71 ±0.23 | 0.84 | 10.42 | 1.58 |
|  | 10-15 | -2.35 ± 1.78 | -5.84 to 1.13 ±0.46 | 0.80 | 7.08 | 2.94 |
|  | 15-20 | -4.56 ± 2.99 | -10.42 to 1.30 ± 0.58 | 0.56 | 7.02 | 5.45 |
|  | 20-25 | -6.10 ± 4.20 | -14.33 to 2.13 ± 0.94 | 0.41 | 5.76 | 7.40 |
|  | 25-30 | -7.28 ± 3.83 | -14.79 to 14.79 ±1.26 | 0.32 | 4.69 | 8.22 |
|  | >30 | -6.99 ± 4.05 | -14.93 to 0.94 ± 2.03 | 0.79 | 6.41 | 8.06 |
| TASK (trials categorized according to performed movement task, all intensities included) | | | | | | |
| mean | Task 1 | -0.53 ± 0.41 | -1.33 to 0.27 ± 0.10 | 0.98 | 6.10 | 0.67 |
|  | Task 2 | -0.50 ± 0.29 | -1.06 to 0.07 ± 0.07 | 0.99 | 3.70 | 0.57 |
|  | Task 3 | -0.53 ± 0.39 | -1.29 to 0.24 ± 0.09 | 0.98 | 5.98 | 0.65 |
|  | Task 4 | -0.60 ± 0.42 | -1.43 to 0.22 ±0.10 | 0.98 | 4.64 | 0.73 |
|  | Task 5 | -0.75 ± 0.53 | -1.78 to 0.28 ±0.12 | 0.99 | 3.43 | 0.92 |
|  | Task 6 | -0.36 ± 0.15 | -0.66 to -0.07 ± 0.06 | 0.97 | 5.14 | 0.39 |
| peak | Task 1 | -5.61 ± 4.57 | -14.57 to 3.36 ± 1.10 | 0.97 | 10.98 | 7.23 |
|  | Task 2 | -2.87 ± 3.30 | -9.33 to 3.59 ± 0.78 | 0.94 | 12.42 | 4.36 |
|  | Task 3 | -3.99 ± 3.25 | -10.36 to 2.38 ± 0.77 | 0.96 | 10.80 | 5.14 |
|  | Task 4 | -3.93 ± 3.42 | -10.64 to 2.77 ± 0.81 | 0.95 | 10.97 | 5.21 |
|  | Task 5 | -4.50 ± 3.35 | -11.08 to 2.07 ± 0.79 | 0.97 | 9.15 | 5.61 |
|  | Task 6 | -4.33 ± 3.23 | -10.66 to 1.99 ± 1.32 | 0.76 | 11.62 | 5.39 |
