# Supplementary material for: Validation of Wearable Sensors during Team Sport-Specific Movements in Indoor Environments
Source: Sensors (Basel). 2019 Aug 7;19(16):3458. doi: 10.3390/s19163458 (PMC6720677; doi:10.3390/s19163458)
Supplement: Supplementary File 1 [file sensors-19-03458-s001.zip › Table S2.docx]

|  |  | MB ± SD | LoA ± CI | r_s_ | CV (%) | RMSE (m·s^-2)^ |
| --- | --- | --- | --- | --- | --- | --- |
| \|acc_vert_\| | | | | | | |
| OVERALL (all trials included) | | | | | | |
| mean | all trials | -0.14 ± 0.16 | -0.47 to 0.18 ± 0.02 | 0.99 | 5.54 | 0.22 |
| peak | all trials | -2.15 ± 2.28 | -6.63 to 2.32 ± 0.23 | 0.97 | 9.23 | 3.14 |
| BALL POSSESSION (all trials included, separated according to ball handling) | | | | | | |
| mean | without ball | -0.14 ± 0.17 | -0.47 to 0.19 ± 0.02 | 0.99 | 5.73 | 0.22 |
|  | with ball | -0.15 ± 0.16 | -0.46 to 0.17 ± 0.02 | 0.99 | 5.35 | 0.22 |
| peak | without ball | -2.22 ± 2.44 | -7.00 to 2.56 ± 0.36 | 0.96 | 9.73 | 3.30 |
|  | with ball | -2.09 ± 2.12 | -6.25 to 2.06 ± 0.30 | 0.97 | 8.73 | 2.98 |
| INTENSITY ( all trials included, separated according to acceleration band) | | | | | | |
| mean | 0-1 | -0.11 ± 0.03 | -0.17 to -0.06 ± 0.02 | 0.95 | 3.14 | 0.11 |
|  | 1-2 | -0.13 ± 0.16 | -0.45 to 0.19 ± 0.02 | 0.99 | 5.62 | 0.21 |
|  | 2-3 | -0.17 ± 0.18 | -0.53 to 0.19 ± 0.02 | 0.98 | 5.62 | 0.25 |
|  | 3-4 | -0.21 ± 0.18 | -0.56 to 0.15 ± 0.03 | 0.97 | 4.44 | 0.28 |
|  | 4-5 | -0.24 ± 0.16 | -0.55 to 0.08 ± 0.04 | 0.98 | 2.81 | 0.29 |
|  | 5-6 | -0.26 v 0.16 | -0.58 to 0.06 ± 0.05 | 0.94 | 2.27 | 0.31 |
|  | >6 | -0.31 ± 0.13 | -0.56 to -0.05 ± 0.05 | 0.84 | 1.97 | 0.33 |
| peak | 0-5 | -0.12 ± 0.30 | -0.70 to 0.46 ± 0.14 | 0.85 | 6.22 | 0.32 |
|  | 5-10 | -0.62 ± 0.58 | -1.75 to 0.51 ± 0.13 | 0.95 | 6.05 | 0.85 |
|  | 10-15 | -1.56 ± 1.42 | -4.35 to 1.23 ± 0.25 | 0.80 | 6.58 | 2.11 |
|  | 15-20 | -3.64 ± 2.21 | -7.97 to 0.69 ± 0.43 | 0.65 | 7.03 | 4.26 |
|  | 20-25 | -4.52 ± 3.36 | -11.10 to 2.05 ± 1.17 | 0.45 | 5.07 | 5.62 |
|  | 25-30 | -4.06 ± 2.90 | -9.74 to 1.63 ± 2.63 | 0.46 | 4.01 | 4.93 |
|  | >30 |  |  |  |  |  |
| TASK (trials categorized according to performed movement task, all intensities included) | | | | | | |
| mean | Task 1 | -0.14 ± 0.15 | -0.43 to 0.15 ± 0.04 | 0.98 | 5.63 | 0.21 |
|  | Task 2 | -0.13 ± 0.10 | -0.32 to 0.06 ± 0.02 | 0.99 | 4.02 | 0.16 |
|  | Task 3 | -0.16 ± 0.16 | -0.48 to 0.17 ± 0.04 | 0.98 | 5.55 | 0.23 |
|  | Task 4 | -0.17 ± 0.18 | -0.53 to 0.19 ± 0.04 | 0.99 | 5.04 | 0.25 |
|  | Task 5 | -0.15 ± 0.19 | -0.53 to 0.23 ± 0.05 | 0.99 | 2.66 | 0.24 |
|  | Task 6 | -0.05 ± 0.18 | -0.40 to 0.31 ± 0.07 | 0.89 | 8.70 | 0.19 |
| peak | Task 1 | -2.12 ± 2.79 | -7.58 to 3.34 ± 0.67 | 0.98 | 10.37 | 3.50 |
|  | Task 2 | -1.35 ± 1.31 | -3.92 to 1.21 ± 0.31 | 0.97 | 6.97 | 1.88 |
|  | Task 3 | -1.99 ± 2.15 | -6.19 to 2.22 ± 0.51 | 0.97 | 8.54 | 2.92 |
|  | Task 4 | -2.34 ± 2.14 | -6.53 to 1.85 ± 0.51 | 0.97 | 8.07 | 3.17 |
|  | Task 5 | -2.91 ± 2.06 | -6.95 to 1.14 ± 0.49 | 0.97 | 7.18 | 3.56 |
|  | Task 6 | -2.34 ± 3.37 | -8.94 to 4.27 ± 1.38 | 0.70 | 11.07 | 4.08 |
| \|acc_hor_\| | | | | | | |
| OVERALL (all trials included) | | | | | | |
| mean | all trials | -0.37 ± 0.32 | -0.99 to 0.25 ± 0.03 | 0.95 | 20.11 | 0.49 |
| peak | All trials | -2.04 ±3.11 | -8.12 to 4.05 ± 0.32 | 0.86 | 30.13 | 3.71 |
| BALL POSSESSION (all trials included, separated according to ball handling) | | | | | | |
| mean | without ball | -0.35 ± 0.35 | -1.04 to 0.34 ± 0.05 | 0.92 | 22.10 | 0.50 |
|  | with ball | -0.39 ± 0.28 | -0.93 to 0.15 ± 0.04 | 0.97 | 17.69 | 0.48 |
| peak | without ball | -0.35 ± 0.35 | -1.04 to 0.34 ± 0.05 | 0.92 | 22.10 | 0.50 |
|  | with ball | -0.39 ± 0.28 | -0.93 to 0.15 ± 0.04 | 0.97 | 17.69 | 0.48 |
| INTENSITY ( all trials included, separated according to acceleration band) | | | | | | |
| mean | 0-1 | -0.49 ± 0.46 | -1.40 to 0.41 ± 0.15 | -0.13 | 22.80 | 0.68 |
|  | 1-2 | -0.36 ± 0.29 | -0.93 to 0.22 ± 0.03 | 0.96 | 12.02 | 0.46 |
|  | 2-3 | -0.41 ± 0.32 | -1.05 to 0.22 ± 0.05 | 0.93 | 8.89 | 0.52 |
|  | 3-4 | -0.47 ± 0.42 | -1.29 to 0.35 ± 0.10 | 0.83 | 7.63 | 0.63 |
|  | 4-5 | -0.70 ± 0.46 | -1.60 to 0.21 ± 0.20 | 0.78 | 4.07 | 0.83 |
|  | 5-6 | -1.32 ± 0.37 | -2.04 to -0.61 ± 0.44 | 0.81 | 3.41 | 1.37 |
|  | >6 |  |  |  |  |  |
| peak | 0-5 | -2.43 ± 2.40 | -7.13 to 2.27 ± 0.80 | 0.57 | 19.12 | 3.41 |
|  | 5-10 | -2.54 ± 2.92 | -8.26 to 3.19 ± 0.49 | 0.47 | 20.27 | 3.87 |
|  | 10-15 | -2.09 ± 3.49 | -8.93 to 4.75 ± 0.71 | 0.38 | 11.48 | 4.06 |
|  | 15-20 | -1.09 ± 2.72 | -6.42 to 4.24 ± 0.69 | 0.38 | 7.39 | 2.92 |
|  | 20-25 | -0.69 ± 3.51 | -7.58 to 6.20 ± 1.26 | 0.42 | 5.74 | 3.56 |
|  | 25-30 | -2.40 ± 2.62 | -7.55 to 2.74 ± 1.90 | 0.51 | 4.69 | 3.52 |
|  | >30 | -3.94 ± 2.14 | -8.13 to 0.25 ± 2.29 | 0.82 | 4.78 | 4.43 |
| TASK (trials categorized according to performed movement task, all intensities included) | | | | | | |
| mean | Task 1 | -0.26 ± 0.19 | -0.63 to 0.11 ± 0.05 | 0.95 | 11.99 | 0.32 |
|  | Task 2 | -0.39 ± 0.28 | -0.95 to 0.16 ± 0.07 | 0.97 | 8.09 | 0.48 |
|  | Task 3 | -0.31 ± 0.17 | -0.66 to 0.03 ± 0.04 | 0.97 | 7.66 | 0.36 |
|  | Task 4 | -0.36 ± 0.23 | -0.81 to 0.08 ± 0.05 | 0.98 | 6.60 | 0.43 |
|  | Task 5 | -0.33 ± 0.38 | -1.06 to 0.41 ± 0.09 | 0.98 | 7.30 | 0.50 |
|  | Task 6 | -0.94 ± 0.45 | -1.83 to -0.06 ± 0.18 | 0.69 | 20.17 | 1.04 |
| peak | Task 1 | -2.95 ± 2.84 | -8.51 to 2.62 ± 0.68 | 0.89 | 26.73 | 4.09 |
|  | Task 2 | -2.14 ± 1.98 | -6.01 to 1.74 ± 0.47 | 0.93 | 17.57 | 2.91 |
|  | Task 3 | -1.52 ± 1.91 | -5.26 to 2.22 ± 0.45 | 0.96 | 19.47 | 2.44 |
|  | Task 4 | -0.59 ± 2.46 | -5.42 to 4.24 ± 0.58 | 0.92 | 18.71 | 2.53 |
|  | Task 5 | -0.85 ± 2.24 | -5.23 to 3.54 ± 0.58 | 0.95 | 15.10 | 2.39 |
|  | Task 6 | -8.48 ± 4.39 | -17.08 to 0.12 ± 1.79 | 0.42 | 42.53 | 9.53 |
| \|acc_res_\| (CF) | | | | | | |
| OVERALL (all trials included) | | | | | | |
| mean | all trials | -0.40 ± 0.29 | -0.98 to 0.17 ± 0.03 | 0.98 | 7.46 | 0.50 |
| peak | All trials | -1.87 ± 2.12 | -6.03 to 2.29 ± 0.22 | 0.97 | 10.63 | 2.83 |
| BALL POSSESSION (all trials included, separated according to ball handling) | | | | | | |
| mean | Without ball | -0.38 ± 0.29 | -0.99 to 0.24 ± 0.05 | 0.98 | 7.46 | 0.50 |
|  | with ball | -0.43 ± 0.27 | -0.96 to 0.11 ± 0.04 | 0.99 | 6.86 | 0.51 |
| peak | Without ball | -1.93 ± 2.27 | -6.38 to 2.52 ± 0.33 | 0.97 | 10.90 | 2.98 |
|  | with ball | -1.81 ± 1.97 | -5.68 to 2.05 ±0.28 | 0.97 | 10.38 | 2.68 |
| INTENSITY ( all trials included, separated according to acceleration band) | | | | | | |
| mean | 0-1 |  |  |  |  |  |
|  | 1-2 | -0.39 ± 0.28 | -0.94 to 0.16 ± 0.03 | 0.98 | 7.64 | 0.48 |
|  | 2-3 | -0.43 ± 0.30 | -1.01 to 0.16 ± 0.03 | 0.98 | 7.43 | 0.52 |
|  | 3-4 | -0.46 ± 0.30 | -1.04 to 0.12 ± 0.04 | 0.97 | 5.88 | 0.54 |
|  | 4-5 | -0.51 ± 0.32 | -1.14 to 0.12 ± 0.05 | 0.95 | 5.71 | 0.60 |
|  | 5-6 | -0.50 ± 0.34 | -1.17 to 0.16 ± 0.08 | 0.96 | 4.70 | 0.61 |
|  | >6 | -0.51 ± 0.26 | -1.21 to 0.19 ± 0.10 | 0.93 | 3.84 | 0.62 |
| peak | 0-5 | -0.53 ± 0.55 | -1.60 to 0.54 ± 0.33 | 0.59 | 9.79 | 0.76 |
|  | 5-10 | -0.96 ± 0.92 | -2.76 to 0.83 ± 0.21 | 0.78 | 11.27 | 1.33 |
|  | 10-15 | -1.12 ± 1.20 | -3.47 to 1.22 ± 0.29 | 0.82 | 6.40 | 1.64 |
|  | 15-20 | -2.02 ± 2.01 | -5.95 to 1.91 ± 0.38 | 0.69 | 6.47 | 2.84 |
|  | 20-25 | -2.83 ± 2.82 | -8.36 to 2.71 ± 0.64 | 0.50 | 5.68 | 3.99 |
|  | 25-30 | -3.03 ± 2.74 | -8.39 to 2.34 ±1.03 | 0.27 | 4.23 | 4.07 |
|  | >30 | -2.91 ± 2.07 | -6.97 to 1.15 ± 1.33 | 0.84 | 5.19 | 3.55 |
| TASK (trials categorized according to performed movement task, all intensities included) | | | | | | |
| mean | Task 1 | -0.29 ± 0.19 | -0.67 to 0.09 ± 0.05 | 0.99 | 5.51 | 0.35 |
|  | Task 2 | -0.44 ± 0.28 | -0.99 to 0.10 ± 0.07 | 0.96 | 6.20 | 0.52 |
|  | Task 3 | -0.36 ± 0.21 | -0.78 to 0.06 ± 0.05 | 0.99 | 4.82 | 0.42 |
|  | Task 4 | -0.43 ± 0.28 | -0.97 to 0.12 ± 0.07 | 0.98 | 4.99 | 0.51 |
|  | Task 5 | -0.37 ± 0.33 | -1.02 to 0.27 ± 0.08 | 0.98 | 3.48 | 0.50 |
|  | Task 6 | -0.75 ± 0.42 | -1.58 to 0.08 ± 0.17 | 0.85 | 10.33 | 0.86 |
| peak | Task 1 | -2.66 ± 2.47 | -7.50 to 2.17 ± 0.59 | 0.99 | 9.80 | 3.62 |
|  | Task 2 | -1.28 ± 1.57 | -4.37 to 1.80 ± 0.37 | 0.95 | 10.50 | 2.03 |
|  | Task 3 | -1.82 ± 1.90 | -5.53 to 1.90 ± 0.45 | 0.98 | 10.19 | 2.62 |
|  | Task 4 | -1.49 ± 1.89 | -5.21 to 2.22 ± 0.45 | 0.96 | 9.98 | 2.41 |
|  | Task 5 | -1.62 ± 2.00 | -5.54 to 2.30 ± 0.47 | 0.96 | 9.00 | 2.57 |
|  | Task 6 | -3.37 ± 2.76 | -8.78 to 2.05 ± 1.13 | 0.77 | 9.71 | 4.34 |
| \|acc_res_\| (KF) | | | | | | |
| OVERALL (all trials included) | | | | | | |
| mean | all trials | -0.66 ± 0.55 | -1.75 to 0.43 ± 0.06 | 0.98 | 8.90 | 0.86 |
| peak | All trials | -3.85 ± 3.52 | -10.74 to 3.05 ± 0.36 | 0.94 | 12.74 | 5.21 |
| BALL POSSESSION (all trials included, separated according to ball handling) | | | | | | |
| mean | with ball | -0.67 ± 0.59 | -1.84 to 0.49 ± 0.09 | 0.98 | 9.09 | 0.90 |
|  | without ball | -0.65 ± 0.51 | -1.65 to 0.36 ± 0.07 | 0.98 | 8.68 | 0.83 |
| peak | With ball | -4.11 ± 3.75 | -11.45 to 3.24 ± 0.55 | 0.95 | 11.97 | 5.56 |
|  | Without ball | -3.60 ± 3.26 | -9.99 to 2.79 ±0.47 | 0.94 | 13.33 | 4.86 |
| INTENSITY ( all trials included, separated according to acceleration band) | | | | | | |
| mean | 0-1 |  |  |  |  |  |
|  | 1-2 | -0.58 ± 0.47 | -1.50 to 0.35 ± 0.05 | 0.97 | 8.67 | 0.75 |
|  | 2-3 | -0.72 ± 0.56 | -1.82 to 0.37 ± 0.06 | 0.97 | 9.13 | 0.91 |
|  | 3-4 | -0.86 ± 0.57 | -1.98 to 0.27 ± 0.07 | 0.94 | 8.94 | 1.03 |
|  | 4-5 | -1.01 ± 0.61 | -2.21 to 0.19 ± 0.10 | 0.91 | 8.95 | 1.18 |
|  | 5-6 | -1.15 ± 0.68 | -2.49 to 0.19 ± 0.16 | 0.87 | 7.95 | 1.34 |
|  | >6 | -1.24 ± 0.70 | -2.60 to 0.13 ± 0.20 | 0.72 | 6.45 | 1.42 |
| peak | 0-5 | -0.60 ± 0.44 | -1.47 to 0.27 ± 0.27 | 0.79 | 6.49 | 0.74 |
|  | 5-10 | -1.22 ± 1.01 | -3.20 to 0.77 ± 0.24 | 0.82 | 10.71 | 1.58 |
|  | 10-15 | -2.63 ± 2.32 | -7.18 to 1.92 ± 0.56 | 0.77 | 7.25 | 3.50 |
|  | 15-20 | -4.44 ± 3.14 | -10.60 to 1.73 ± 0.60 | 0.49 | 7.79 | 5.44 |
|  | 20-25 | -5.63 ± 4.14 | -13.74 to 2.48 ± 0.94 | 0.39 | 6.01 | 6.98 |
|  | 25-30 | -6.63 ± 3.74 | -13.95 to 0.70 ± 1.41 | 0.22 | 4.30 | 7.60 |
|  | >30 | -7.00 ± 3.61 | -14.08 to 0.08 ± 2.32 | 0.85 | 5.89 | 7.85 |
| TASK (trials categorized according to performed movement task, all intensities included) | | | | | | |
| mean | Task 1 | -0.61 ± 0.51 | -1.61 to 0.39 ± 0.12 | 0.95 | 9.79 | 0.79 |
|  | Task 2 | -0.58 ± 0.44 | -1.45 to 0.29 ± 0.10 | 0.96 | 6.73 | 0.73 |
|  | Task 3 | -0.60 ± 0.50 | -1.58 to 0.38 ± 0.12 | 0.96 | 8.87 | 0.78 |
|  | Task 4 | -0.70 ± 0.55 | -1.78 to 0.39 ± 0.13 | 0.95 | 7.56 | 0.89 |
|  | Task 5 | -0.90 ± 0.73 | -2.32 to 0.52 ± 0.17 | 0.91 | 6.33 | 1.15 |
|  | Task 6 | -0.40 ± 0.18 | -0.76 to -0.04 ± 0.07 | 0.94 | 6.10 | 0.44 |
| peak | Task 1 | -5.32 ± 4.48 | -14.10 to 3.47 ± 1.08 | 0.97 | 11.57 | 6.95 |
|  | Task 2 | -2.59 ± 2.93 | -8.33 to 3.15 ± 0.69 | 0.91 | 13.80 | 3.90 |
|  | Task 3 | -3.73 ± 3.06 | -9.74 to 2.27 ± 0.73 | 0.96 | 10.67 | 4.82 |
|  | Task 4 | -3.53 ± 3.18 | -9.76 to 2.71 ± 0.75 | 0.95 | 11.07 | 4.75 |
|  | Task 5 | -4.02 ± 3.25 | -10.39 to 2.35 ± 0.77 | 0.96 | 9.53 | 5.17 |
|  | Task 6 | -4.14 ± 3.41 | -10.83 to 2.56 ± 1.40 | 0.69 | 11.18 | 5.35 |
