# Supplementary material for: Validation of Wearable Sensors during Team Sport-Specific Movements in Indoor Environments
Source: Sensors (Basel). 2019 Aug 7;19(16):3458. doi: 10.3390/s19163458 (PMC6720677; doi:10.3390/s19163458)
Supplement: Supplementary File 1 [file sensors-19-03458-s001.zip › Table S3.docx]

|  |  | MB ± SD | LoA ± CI | r_s_ | CV (%) | RMSE (m·s^-2)^ |
| --- | --- | --- | --- | --- | --- | --- |
| \|acc_vert_\| | | | | | | |
| OVERALL (all trials included) | | | | | | |
| mean | all trials | -0.12 ± 0.15 | -0.40 to 0.17 ± 0.02 | 0.97 | 10.57 | 0.19 |
| peak | all trials | -0.34 ± 0.84 | -1.99 to 1.32 ± 0.09 | 0.95 | 12.08 | 0.91 |
| BALL POSSESSION (all trials included, separated according to ball handling) | | | | | | |
| mean | without ball | -0.10 ± 0.15 | -0.40 to 0.19 ± 0.02 | 0.98 | 9.96 | 0.18 |
|  | with ball | -0.13 ± 0.15 | -0.41 to 0.16 ± 0.02 | 0.95 | 11.11 | 0.19 |
| peak | without ball | -0.25 ± 0.90 | -2.01 to 1.51 ± 0.13 | 0.95 | 12.18 | 0.93 |
|  | with ball | -0.43 ± 0.78 | -1.96 to 1.10 ± 0.11 | 0.95 | 11.80 | 0.89 |
| INTENSITY ( all trials included, separated according to acceleration band) | | | | | | |
| mean | 0-1 | -0.15 ± 0.11 | -0.37 to 0.06 ± 0.03 | 0.76 | 10.79 | 0.19 |
|  | 1-2 | -0.11 ± 0.15 | -0.41 to 0.19 ± 0.02 | 0.95 | 9.62 | 0.19 |
|  | 2-3 | -0.09 ± 0.16 | -0.40 to 0.22 ± 0.03 | 0.96 | 5.93 | 0.18 |
|  | 3-4 | -0.07 ± 0.15 | -0.37 to 0.23 ±0.06 | 0.88 | 4.63 | 0.17 |
|  | 4-5 | -0.02 ± 0.14 | -0.30 to 0.25 ± 0.14 | 0.84 | 2.65 | 0.14 |
|  | 5-6 |  |  |  |  |  |
|  | >6 |  |  |  |  |  |
| peak | 0-5 | -0.25 ± 0.56 | -1.34 to 0.84 ± 0.10 | 0.89 | 10.91 | 0.61 |
|  | 5-10 | -0.50 ± 0.83 | -2.13 to 1.13 ± 0.11 | 0.85 | 9.94 | 0.97 |
|  | 10-15 | 0.60 ± 1.19 | -1.73 to 2.93 ± 0.44 | 0.37 | 10.05 | 1.32 |
|  | 15-20 |  |  |  |  |  |
|  | 20-25 |  |  |  |  |  |
|  | 25-30 |  |  |  |  |  |
|  | >30 |  |  |  |  |  |
| TASK (trials categorized according to performed movement task, all intensities included) | | | | | | |
| mean | Task 1 | -0.12 ± 0.12 | -0.37 to 0.12 ± 0.03 | 0.96 | 10.73 | 0.17 |
|  | Task 2 | -0.14 ± 0.13 | -0.41 to 0.12 ± 0.03 | 0.96 | 9.52 | 0.20 |
|  | Task 3 | -0.13 ± 0.16 | -0.44 to 0.17 ± 0.04 | 0.93 | 13.53 | 0.21 |
|  | Task 4 | -0.13 ± 0.14 | -0.42 to 0.15 ± 0.03 | 0.95 | 9.47 | 0.20 |
|  | Task 5 | -0.07 ± 0.13 | -0.33 to 0.19 ±0.03 | 0.99 | 4.55 | 0.15 |
|  | Task 6 | -0.03 ± 0.20 | -0.43 to 0.37 ± 0.08 | 0.83 | 11.99 | 0.21 |
| peak | Task 1 | -0.42 ± 0.69 | -1.77 to 0.93 ± 0.17 | 0.94 | 11.88 | 0.81 |
|  | Task 2 | -0.24 v 0.68 | -1.57 to 1.10 ± 0.16 | 0.93 | 11.09 | 0.72 |
|  | Task 3 | -0.36 ± 0.75 | -1.83 to 1.11 ± 0.18 | 0.94 | 12.61 | 0.83 |
|  | Task 4 | -0.49 ± 0.91 | -2.28 to 1.29 ± 0.22 | 0.91 | 11.90 | 1.03 |
|  | Task 5 | -0.50 ± 0.79 | -2.06 to 1.05 ± 0.19 | 0.92 | 9.17 | 0.94 |
|  | Task 6 | 0.63 ± 1.19 | -1.70 to 2.95 ± 0.48 | 0.40 | 10.49 | 1.33 |
| \|acc_hor_\| | | | | | | |
| OVERALL (all trials included) | | | | | | |
| mean | all trials | -0.32 ±0.32 | -0.95 to 0.30 ± 0.03 | 0.91 | 29.13 | 0.46 |
| peak | All trials | -0.89 ± 2.67 | -6.13 to 4.35 ± 0.27 | 0.75 | 43.21 | 2.81 |
| BALL POSSESSION (all trials included, separated according to ball handling) | | | | | | |
| mean | without ball | -0.29 ±0.36 | -0.99 to 0.41 ± 0.05 | 0.87 | 31.48 | 0.46 |
|  | with ball | -0.36 ± 0.28 | -0.90 to 0.19 ± 0.04 | 0.95 | 26.17 | 0.45 |
| peak | without ball | -0.96 ± 3.01 | -6.86 to 4.95 ± 0.44 | 0.73 | 45.84 | 3.16 |
|  | with ball | -0.82 ± 2.30 | -5.33 to 3.69 ± 0.33 | 0.77 | 40.45 | 2.44 |
| INTENSITY ( all trials included, separated according to acceleration band) | | | | | | |
| mean | 0-1 | -0.42 ± 0.42 | -1.23 to 0.40 ± 0.09 | 36.05 | 6.34 | 0.59 |
|  | 1-2 | -0.30 ± 0.28 | -0.85 to 0.26 ± 0.03 | 12.99 | 6.78 | 0.41 |
|  | 2-3 | -0.36 ± 0.38 | -1.10 to 0.39 ± 0.08 | 10.39 | 3.50 | 0.52 |
|  | 3-4 | -0.49 ± 0.46 | -1.40 to 0.42 ±0.20 | 4.50 | 1.31 | 0.67 |
|  | 4-5 |  |  |  |  |  |
|  | 5-6 |  |  |  |  |  |
|  | >6 |  |  |  |  |  |
| peak | 0-5 | -2.20 ± 3.06 | -8.20 to 3.81 ± 0.54 | 0.33 | 27.60 | 3.77 |
|  | 5-10 | -0.60 ± 2.13 | -4.78 to 3.58 ± 0.34 | 0.59 | 18.91 | 2.21 |
|  | 10-15 | 0.53 ± 2.19 | -3.76 to 4.82 ± 0.48 | 0.40 | 11.40 | 2.25 |
|  | 15-20 | -0.57 ± 1.45 | -3.41 to 2.26 ± 0.69 | 0.55 | 5.85 | 1.54 |
|  | 20-25 |  |  |  |  |  |
|  | 25-30 |  |  |  |  |  |
|  | >30 |  |  |  |  |  |
| TASK (trials categorized according to performed movement task, all intensities included) | | | | | | |
| mean | Task 1 | -0.24 ± 0.18 | -0.59 to 0.11 ± 0.04 | 0.91 | 15.44 | 0.30 |
|  | Task 2 | -0.33 ± 0.28 | -0.89 to 0.22 ±0.07 | 0.95 | 10.45 | 0.44 |
|  | Task 3 | -0.28 ± 0.18 | -0.64 to 0.07 ± 0.04 | 0.95 | 11.14 | 0.34 |
|  | Task 4 | -0.31 ± 0.23 | -0.76 to 0.13 ± 0.05 | 0.95 | 9.08 | 0.39 |
|  | Task 5 | -0.23 ± 0.33 | -0.88 to 0.43 ± 0.08 | 0.97 | 9.51 | 0.40 |
|  | Task 6 | -0.99 ± 0.47 | -1.91 to -0.07 ± 0.19 | 0.65 | 24.10 | 1.10 |
| peak | Task 1 | -1.37 ± 1.60 | -4.50 to 1.76 ± 0.38 | 0.85 | 30.09 | 2.10 |
|  | Task 2 | -1.17 ± 1.67 | -4.44 to 2.11 ± 0.39 | 0.91 | 19.98 | 2.04 |
|  | Task 3 | -0.22 ± 1.58 | -3.31 to 2.87 ± 0.37 | 0.90 | 25.85 | 1.59 |
|  | Task 4 | 0.44 ± 1.66 | -2.81 to 3.69 ± 0.39 | 0.92 | 18.64 | 1.71 |
|  | Task 5 | 0.26 ± 1.60 | -2.87 to 3.40 ± 0.38 | 0.93 | 17.66 | 1.62 |
|  | Task 6 | -8.08 ± 3.33 | -14.61 to -1.56 ± 1.36 | 0.47 | 33.14 | 8.73 |
| \|acc_res_\| (CF) | | | | | | |
| OVERALL (all trials included) | | | | | | |
| mean | all trials | -0.33 ±0.29 | -0.91 to 0.24 ± 0.03 | 0.96 | 10.78 | 0.44 |
| peak | All trials | -0.14 ±1.40 | -2.88 to 2.60 ± 0.14 | 0.95 | 15.98 | 1.40 |
| BALL POSSESSION (all trials included, separated according to ball handling) | | | | | | |
| mean | Without ball | -0.30 ± 0.31 | -0.90 to 0.31 ± 0.05 | 0.96 | 11.02 | 0.43 |
|  | with ball | -0.37 ± 0.28 | -0.91 to 0.17 ± 0.04 | 0.96 | 10.34 | 0.46 |
| peak | Without ball | -0.15 ± 1.37 | -2.85 to 2.54 ± 0.20 | 0.96 | 15.27 | 1.38 |
|  | with ball | -0.13 ± 1.42 | -2.92 to 2.66 ± 0.20 | 0.93 | 16.67 | 1.43 |
| INTENSITY ( all trials included, separated according to acceleration band) | | | | | | |
| mean | 0-1 | -0.25 ± 0.07 | -0.39 to -0.11 ±0.08 | -0.10 | 2.62 | 0.26 |
|  | 1-2 | -0.33 ± 0.30 | -0.91 to 0.24 ± 0.03 | 0.96 | 10.78 | 0.45 |
|  | 2-3 | -0.35 ± 0.32 | -0.98 to 0.27 ± 0.04 | 0.92 | 9.97 | 0.48 |
|  | 3-4 | -0.35 ± 0.39 | -1.11 to 0.40 ± 0.08 | 0.90 | 9.11 | 0.52 |
|  | 4-5 | -0.28 ± 0.37 | -1.01 to 0.45 ± 0.11 | 0.82 | 5.17 | 0.46 |
|  | 5-6 | -0.31 ± 0.42 | -1.12 to 0.51 ± 0.25 | 0.50 | 3.92 | 0.51 |
|  | >6 |  |  |  |  |  |
| peak | 0-5 | -0.65 ± 0.71 | -2.05 to 0.74 ± 0.18 | 0.71 | 14.75 | 0.96 |
|  | 5-10 | -0.32 ± 1.25 | -2.77 to 2.14 ± 0.21 | 0.81 | 12.94 | 1.29 |
|  | 10-15 | 0.14 ± 1.72 | -3.23 to 3.51 ± 0.30 | 0.62 | 9.60 | 1.72 |
|  | 15-20 | 0.46 ± 1.10 | -1.69 to 2.61 ± 0.37 | 0.84 | 4.60 | 1.18 |
|  | 20-25 | -0.24 ± 1.63 | -3.43 to 2.95 ± 2.76 | 0.00 | 1.59 | 1.43 |
|  | 25-30 |  |  |  |  |  |
|  | >30 |  |  |  |  |  |
| TASK (trials categorized according to performed movement task, all intensities included) | | | | | | |
| mean | Task 1 | -0.27 ± 0.17 | -0.61 to 0.07 ± 0.04 | 0.95 | 8.86 | 0.32 |
|  | Task 2 | -0.39 ± 0.30 | -0.98 to 0.20 ± 0.07 | 0.93 | 8.90 | 0.49 |
|  | Task 3 | -0.31 ± 0.21 | -0.72 to 0.10 ±0.05 | 0.95 | 8.58 | 0.38 |
|  | Task 4 | -0.35 ± 0.26 | -0.85 to 0.16 ± 0.06 | 0.95 | 7.25 | 0.43 |
|  | Task 5 | -0.22 ± 0.31 | -0.83 to 0.40 ± 0.07 | 0.95 | 5.86 | 0.38 |
|  | Task 6 | -0.73 ± 0.41 | -1.53 to 0.06 ±0.17 | 0.76 | 12.80 | 0.84 |
| peak | Task 1 | -0.50 ± 1.14 | -2.74 to 1.74 ± 0.28 | 0.90 | 17.16 | 1.25 |
|  | Task 2 | -0.58 ± 1.41 | -3.35 to 2.19 ± 0.33 | 0.91 | 16.68 | 1.53 |
|  | Task 3 | 0.04 ± 1.14 | -2.20 to 2.28 ± 0.27 | 0.95 | 15.32 | 1.14 |
|  | Task 4 | 0.47 ± 1.12 | -1.73 to 2.67 ±0.27 | 0.96 | 11.41 | 1.21 |
|  | Task 5 | 0.39 ± 1.14 | -1.85 to 2.63 ± 0.27 | 0.96 | 11.39 | 1.20 |
|  | Task 6 | -1.74 ± 2.06 | -5.79 to 2.31 ± 0.84 | 0.61 | 10.42 | 2.69 |
| \|acc_res_\| (KF) | | | | | | |
| OVERALL (all trials included) | | | | | | |
| mean | all trials | -2.00 ± 1.23 | -4.41 to 0.40 ± 0.13 | 0.90 | 16.48 | 2.35 |
| peak | All trials | -3.25 ±2.85 | -8.83 to 2.33 ± 0.29 | 0.85 | 22.56 | 4.32 |
| BALL POSSESSION (all trials included, separated according to ball handling) | | | | | | |
| mean | with ball | -2.03 ± 1.29 | -4.56 to 0.50 ± 0.19 | 0.90 | 17.85 | 2.40 |
|  | without ball | -1.99 ± 1.16 | -4.25 to 0.28 ± 0.17 | 0.90 | 14.99 | 2.30 |
| peak | With ball | -3.12 ± 2.98 | -8.97 to 2.72 ± 0.44 | 0.85 | 22.90 | 4.32 |
|  | Without ball | -3.39 ± 2.71 | -8.69 to 1.92 ± 0.39 | 0.86 | 21.89 | 4.33 |
| INTENSITY ( all trials included, separated according to acceleration band) | | | | | | |
| mean | 0-1 | -0.79 ± 0.71 | -2.19 to 0.61 ± 0.81 | -0.15 | 2.63 | 1.04 |
|  | 1-2 | -2.01 ± 1.22 | -4.41 to 0.38 ± 0.13 | 0.90 | 16.24 | 2.36 |
|  | 2-3 | -2.49 ± 1.17 | -4.78 to -0.21 ± 0.15 | 0.83 | 14.53 | 2.75 |
|  | 3-4 | -3.40 ± 1.14 | -5.64 to -1.16 ± 0.24 | 0.87 | 10.56 | 3.59 |
|  | 4-5 | -4.06 ± 0.94 | -5.91 to -2.21 ± 0.28 | 0.66 | 6.92 | 4.17 |
|  | 5-6 | -4.09 ± 0.65 | -5.36 to -2.82 ±0.39 | 0.26 | 4.05 | 4.14 |
|  | >6 |  |  |  |  |  |
| peak | 0-5 | -1.34 ± 1.51 | -4.29 to 1.62 ± 0.37 | 0.72 | 15.95 | 2.01 |
|  | 5-10 | -3.93 ± 2.94 | -9.69 to 1.83 ± 0.49 | 0.70 | 14.54 | 4.90 |
|  | 10-15 | -3.36 v 2.83 | -8.91 to 2.19 ± 0.49 | 0.36 | 11.58 | 4.39 |
|  | 15-20 | -3.64 ± 2.92 | -9.37 to 2.09 ± 1.00 | 0.69 | 6.10 | 4.66 |
|  | 20-25 | -2.51 ± 1.99 | -6.41 to 1.39 ± 3.38 | 0.80 | 1.49 | 3.04 |
|  | 25-30 |  |  |  |  |  |
|  | >30 |  |  |  |  |  |
| TASK (trials categorized according to performed movement task, all intensities included) | | | | | | |
| mean | Task 1 | -1.68 ± 0.97 | -3.58 to 0.22 ± 0.23 | 0.78 | 18.65 | 1.94 |
|  | Task 2 | -1.73 ± 0.84 | -3.38 to -0.08 ± 0.20 | 0.94 | 8.73 | 1.92 |
|  | Task 3 | -1.78 ± 0.96 | -3.66 to 0.09 ± 0.23 | 0.87 | 15.21 | 2.02 |
|  | Task 4 | -2.11 ± 1.07 | -4.20 to -0.02 ± 0.25 | 0.92 | 9.84 | 2.36 |
|  | Task 5 | -3.08 ± 1.58 | -6.18 to 0.02 ± 0.37 | 0.90 | 7.71 | 3.46 |
|  | Task 6 | -0.93 ± 0.29 | -1.50 to -0.35 ± 0.12 | 0.88 | 8.69 | 0.97 |
| peak | Task 1 | -4.88 ± 3.46 | -11.66 to 1.91 ± 0.83 | 0.85 | 20.02 | 5.97 |
|  | Task 2 | -2.58 ± 2.40 | -7.29 to 2.13 ± 0.57 | 0.85 | 17.82 | 3.52 |
|  | Task 3 | -3.56 ± 2.98 | -9.39 to 2.28 ± 0.71 | 0.88 | 19.22 | 4.63 |
|  | Task 4 | -2.23 ± 1.92 | -5.99 to 1.54 ± 0.45 | 0.92 | 14.95 | 2.94 |
|  | Task 5 | -2.70 ± 2.21 | -7.02 to 1.63 ± 0.52 | 0.96 | 12.23 | 3.48 |
|  | Task 6 | -4.46 ± 3.22 | -10.78 to 1.86 ±1.32 | -0.04 | 13.25 | 5.49 |
